# Assessment of smell disturbances 6 months after COVID-19 in Polish population

**DOI:** 10.1038/s41598-024-62114-y

**Published:** 2024-05-16

**Authors:** Jakub Okrzeja, Sebastian Sołomacha, Maciej Alimowski, Paweł Sowa, Marlena Dubatówka, Magda Łapińska, Łukasz Kiszkiel, Łukasz Szczerbiński, Piotr Paweł Laskowski, Piotr Czupryna, Bożena Kubas, Adam Garkowski, Karol Adam Kamiński, Anna Moniuszko-Malinowska

**Affiliations:** 1 Medical University of Bialystok, Żurawia 14, 15-540 Bialystok, Poland; 2 Department of Population Medicine and Lifestyle Diseases Prevention, Medical University of Bialystok, Białystok, Poland; 3 Doctoral School of Social Sciences, University of Bialystok, Białystok, Poland; 4 Society and Cognition Unit, University of Bialystok, Białystok, Poland; 5 Clinical Research Centre, Medical University of Bialystok, Białystok, Poland; 6 Department of Endocrinology, Diabetology and Internal Medicine, Medical University of Bialystok, Białystok, Poland; 7 Department of Infectious Diseases and Neuroinfections, Medical University of Bialystok, Białystok, Poland; 8 Department of Radiology, Medical University of Bialystok, Białystok, Poland

**Keywords:** Viral infection, SARS-CoV-2, Infectious-disease diagnostics, Infection

## Abstract

Considering the frequency and severity of olfactory disorders associated with SARS-CoV-2 infection, attention to the olfactory loss has expanded. The aim of our study was to assess of smell disturbances 6 months after COVID-19. The study population consisted of 2 groups: 196 Post-COVID-19 patients who were hospitalized because of COVID-19, control sample–130 patients without reported smell disorders from general population-Bialystok PLUS study. People from both groups were asked to participate in the Sniffin Sticks Test (half year after the disease). Sniffin Sticks Test consisted of 12 standardized smell samples. The participant's test score was counted based on correct scent recognition. Middle/older age was related with lower likelihood of olfaction recovery. The biggest differences in recognition of particular fragrances were observed for: orange and lemon, lemon and coffee (p.adj < 0.001). Patients had the greatest problem in assessing smell of lemon. The comparison of scores between Delta, Omicron, Wild Type, Wild Type Alpha waves showed statistically significant difference between Delta and Wild Type waves (p = 0.006). Duration of the disease (r = 0.218), age (r = -0.253), IL-6 (r = -0.281) showed significant negative correlations with the score. Statistically significant variables in the case of smell disorders were Omicron wave (CI = 0.045–0.902; P = 0.046) and Wild Type wave (CI = 0.135–0.716; P = 0.007) compared to Delta wave reference. Moreover, patients with PLT count below 150 000/μl had greater olfactory disorders than those with PLT count over 150 000/μl. There are: smell differences between post-COVID-19 patients and healthy population; statistically significant difference between Delta and Wild Type waves in Post-COVID-19 group in score of the Sniffin Sticks Test. Smell disturbances depend on the age, cognitive impairments, clinical characteristics of the COVID-19 disease and sex of the patient.

## Introduction

The coronavirus disease 2019 (COVID-19) pandemic was caused by the severe acute respiratory syndrome coronavirus 2 (SARS-CoV-2)^1^. Moreover, the first cases of this disease were reported in Wuhan, Hubei Province, China^2^. SARS-CoV-2 is usually associated with pulmonary infection which leads to pneumonia, but recent studies demonstrate that other organs may be affected e.g., in the gastrointestinal, cardiovascular, nervous, and immune systems^3,4^.

An olfactory loss after viral infection is well-documented and the viruses responsible for this condition are adenovirus, influenza virus, rhinovirus, and coronavirus^5,6^. Considering the frequency and severity of olfactory impairment connected with SARS-CoV-2 infection, attention to the olfactory loss after viral illness has expanded significantly due to the COVID-19 pandemic. Furthermore, SARS-CoV-2 associated olfactory dysfunction is rarely connected with the nasal obstruction or rhinorrhea compared to other respiratory viruses causing olfactory loss^7^. The incidence of acute olfactory loss in acute phase of COVID-19 ranges from 34 to 86%^8–10^. After 2 weeks of recovery following COVID-19, an estimated 44% to 64% of these individuals recover olfaction^7,10^. In addition, some studies described recovery from initial COVID-19 for up to half year^11–16^. For example, Petrocelli et al. studied that 6 months after the onset of the disease, approximately 6% of patients still have a severe persistent olfactory disorders. The functional recovery is most common in the first two months in relation to smell and after this time, the likelihood of improvement is significantly decreased^11^.

Some theories have been described to explain the pathogenesis of COVID-19-associated anosmia, including oedema of the olfactory cleft mucous membrane, damage of olfactory epithelium either within the olfactory receptor cells or the supporting non-neural cells, damage to the olfactory bulb, and impairment of the central olfactory pathways^17^. Unfortunately, the pathogenesis of COVID-19-associated anosmia is still not fully explained. It seems to be due to sensorineural damage, with infection of the olfactory epithelium support cells via the angiotensin-converting enzyme 1 receptor and disorder of the olfactory epithelium caused by inflammatory process, and probably with direct olfactory sensory neurons infection mediated by the neuropilin-1 receptor^17^. It might also be related to genetic variables, involvement of the higher olfactory pathways and a conductive component of olfactory disorders^17^.

In our study we aimed to assess the smell disturbances six months after COVID-19 using Sniffin Sticks Test.

## Material and methods

### The study population consisted of 2 groups

- *Group I* Post-COVID-19 group–196 patients, mean age—53.66 ± 12.48 years, who were hospitalized for COVID-19 after the positive SARS-CoV-2 real-time polymerase chain reaction (RT-PCR) or antigen test. They were assessed approximately 6 months after the infection. Those patients suffered from chronic diseases: 95 (48.47%) from hypertension, 99 (50.51%) from obesity (BMI > 30), 21 (10.71%) from cancer. 37 of them were taking β-blockers (18.88%), 22 were taking anticoagulants (11.22%), and 28 were taking drugs that reduce cholesterol and triglyceride levels in the blood (14.29%).
- *Group II* control group–130 patients, mean age—64.87 ± 15.32 years, without smell disturbances from a population study cohort—Bialystok PLUS. Those patients suffered from chronic diseases: 68 (52.31%) from hypertension, 49 (37.69%) from obesity (BMI > 30), 19 (14.62%) from diabetes. 41 of them were taking β-blockers (31.54%), 38 were taking anticoagulants (29.23%), 43 were taking drugs that reduce cholesterol and triglyceride levels in the blood (33.08%), 19 were taking oral antidiabetic drugs (14.62%), 25 were taking angiotensin-converting enzyme inhibitors (19.23%), 17 were taking diuretics (13.08%), 17 were taking drugs used in peptic ulcer disease and gastroesophageal reflux (13.08%), and 15 were taking drugs used in hypothyroidism (11.54%).

The Bialystok PLUS study describes the health of the local community by analysis of the examinations and questionnaires of a carefully selected cohort representative for the local population. It is being conducted from 2018 on a sample of randomly selected Bialystok residents aged 20–80 years old^18^.

Patients were selected according to sex, age and wave number. Patients were selected based on anti-N negative results.

We assessed the patients 6 months after the infection after each wave of pandemics. Particular variant of the virus was assessed by PCR method. The variant PCR testing was performed in the local population and it was described in: “RT-COVAR map: Monitoring of SARS-COV-2 variants and mutations in Poland”^19^. RT-COVAR map is a database containing data on SARS-CoV-2 variants from the epidemiological surveillance of COVID-19 in Poland collected by the State Sanitary Inspection and the National Institute of Public Health-National Research Institute^19^. Worldometers summarized the waves of COVID-19 in Poland as:

- Wave 1—from 29.02.2020 to 31.12.2020–Wild Type variants.
- Wave 2—from 01.01.2021 to end of April 2021–Wild Type Alpha variants.
- Wave 3—from 01.05.2021 to 31.12.2021–Delta variants.
- Wave 4—from 01.01.2022 to 31.03.2022–Omicron variants^20^.

**Figure 1:**
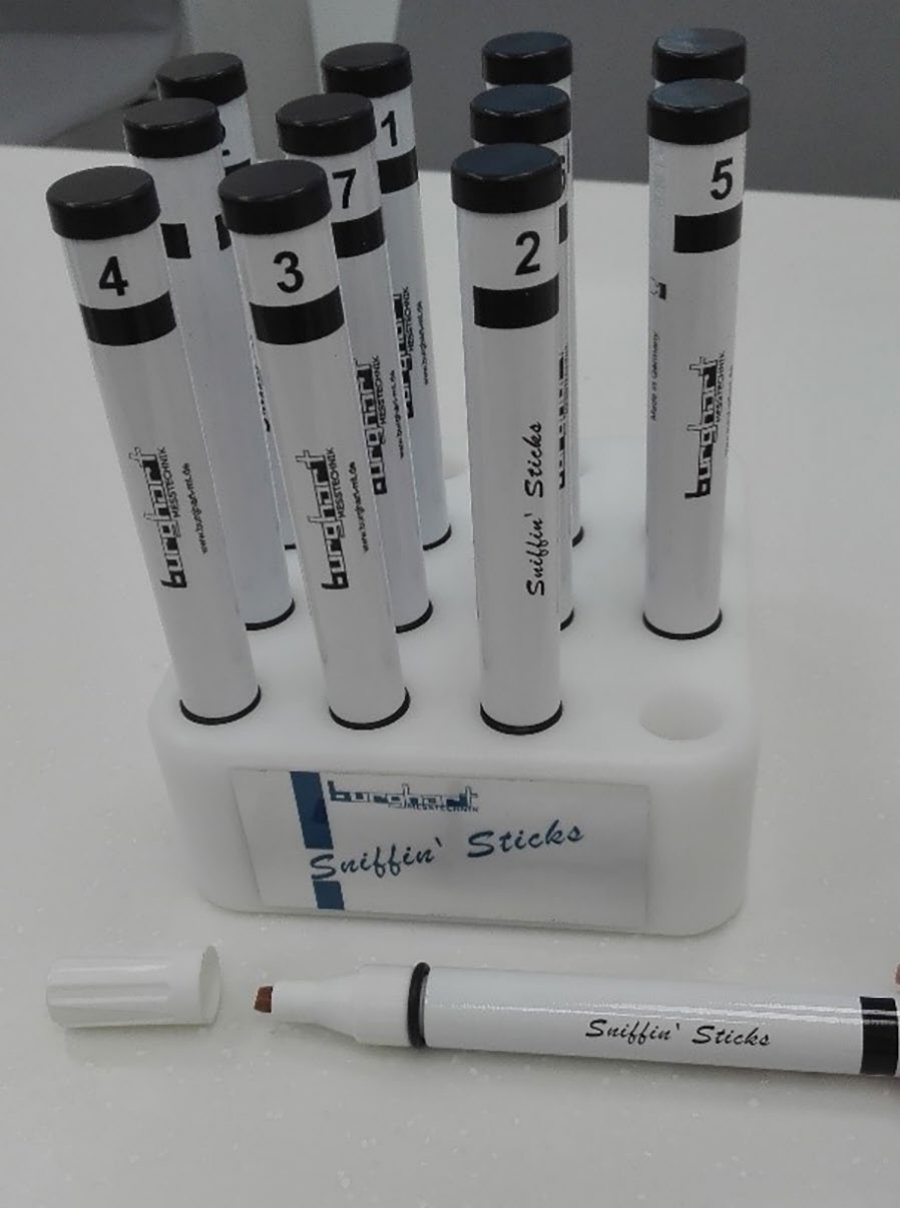
Sniffin Sticks.

People from both groups were asked to participate in the Sniffin Sticks Test. Sniffin Sticks Test is an examination used to assess olfactory disorders. This test consists of 12 reusable standardized fragrance samples (food and non-food smells). The fragrances in our study's samples are: 1. Orange; 2. Leather; 3. Cinnamon; 4. Mint; 5. Banana; 6. Lemon; 7. Licorice; 8. Coffee; 9. Cloves; 10. Pineapple; 11. Rose; 12. Fish. For the test, sticks with a material soaked in a fragrance are used (Fig. 1). After removing the cap, the tip of the stick is placed in front of the participant's nostrils. The proband must not touch his nose to the stick. The Sniffin Sticks come with 12 answer cards. There are 4 answers on each card. The participant chooses one scent from the list presented to him. The person conducting the test writes whether the participant correctly recognized the smell. The participant's test score was counted based on correct scent recognition and could range from 0 to 12.

### Statistical methods

Statistical analyses were performed using the R programming language^21^. All necessary data transformations were performed using “tidyverse” package^22^, “ggstatsplot” package for visualizations of statistical tests^23^. Statistical significance was determined using a significance level of α = 0.05, where a p-value below this threshold was considered important. To compare the differences, the Kruskal–Wallis test was utilized. In addition, a post-hoc analysis was conducted using the Dunn test to perform pairwise comparisons among multiple groups. The resulting p-values were adjusted using the Bonferroni correction, and significant differences were identified if the adjusted p-value was below 0.05. In addition to group comparisons, the relationship between continuous variables was examined using Pearson's correlation coefficient and Dunn test for relationship between continuous variables and factors. Quasi-Poisson and Logistic Regression models were used for statistical inference. Propensity score weighting using optimal full weighting was used to mitigate the difference in age between Group I and Group II. Propensity scores were estimated using a logistic regression of the treatment on the covariates. Age was used as the only covariate. Average effect of the treatment (ATT) was the target estimand. After weighting, all standardized mean differences for the covariates were below 0.01, indicating adequate balance. Full weighting uses all treated and all control units, so no units were discarded.

### Ethical approval statement

This study was conducted according to the guidelines of the Declaration of Helsinki, and approved by the Bioethical Committee of Medical University of Bialystok (Poland) on 26 November 2020 (approval number: APK.002.346.2020).

### Informed consent statement

Informed consent was obtained from all subjects involved in the study.

## Results

### Baseline characteristics

In Group I the mean age was 53.7 years (standard deviation = 12.6). 51% individuals were male, 49% were female. The mean age of the patients in Group II was 64.9 years (standard deviation = 12.8). In terms of sex distribution in control sample, 55% individuals were male and 45% individuals were female.

The study participants were identified in different waves of the COVID-19 pandemic. In Group I 87 (44.4%) of them were identified in Delta wave, 11 (5.6%) in Omicron wave, 72 (36.7%) in Wild Type wave, and 26 (13.3%) in Wilde Type Alpha wave. In Group II 30 (23.1%) of them were identified in Delta wave, 78 (60%) in Omicron wave, 1 (0.8%) in Wild Type wave, and 21 (16.2%) in Wilde Type Alpha wave.

### Olfactory function according to clinical outcomes

Propensity score weights estimated with logistic regression were used to mitigate the difference in age between Group I and Group II. The results are presented in Table 1.

**Table 1:** Regression model of Sniffin Sticks Test total score in Group I and Group II.

Predictors	Estimates	std. Error	CI	*p*
(Intercept)	11.299	0.570	10.178–12.422	< 0.001
Group I	Reference			
Group II	0.483	0.220	0.049–0.916	0.029
Age	− 0.039	0.009	− 0.055–0.022	< 0.001
Observations	325			
R^2^/R^2^ adjusted	0.073 / 0.067			

Ordinary least squares regression.

### Sniffin Sticks Test results for control sample and research sample

**Figure 2:**
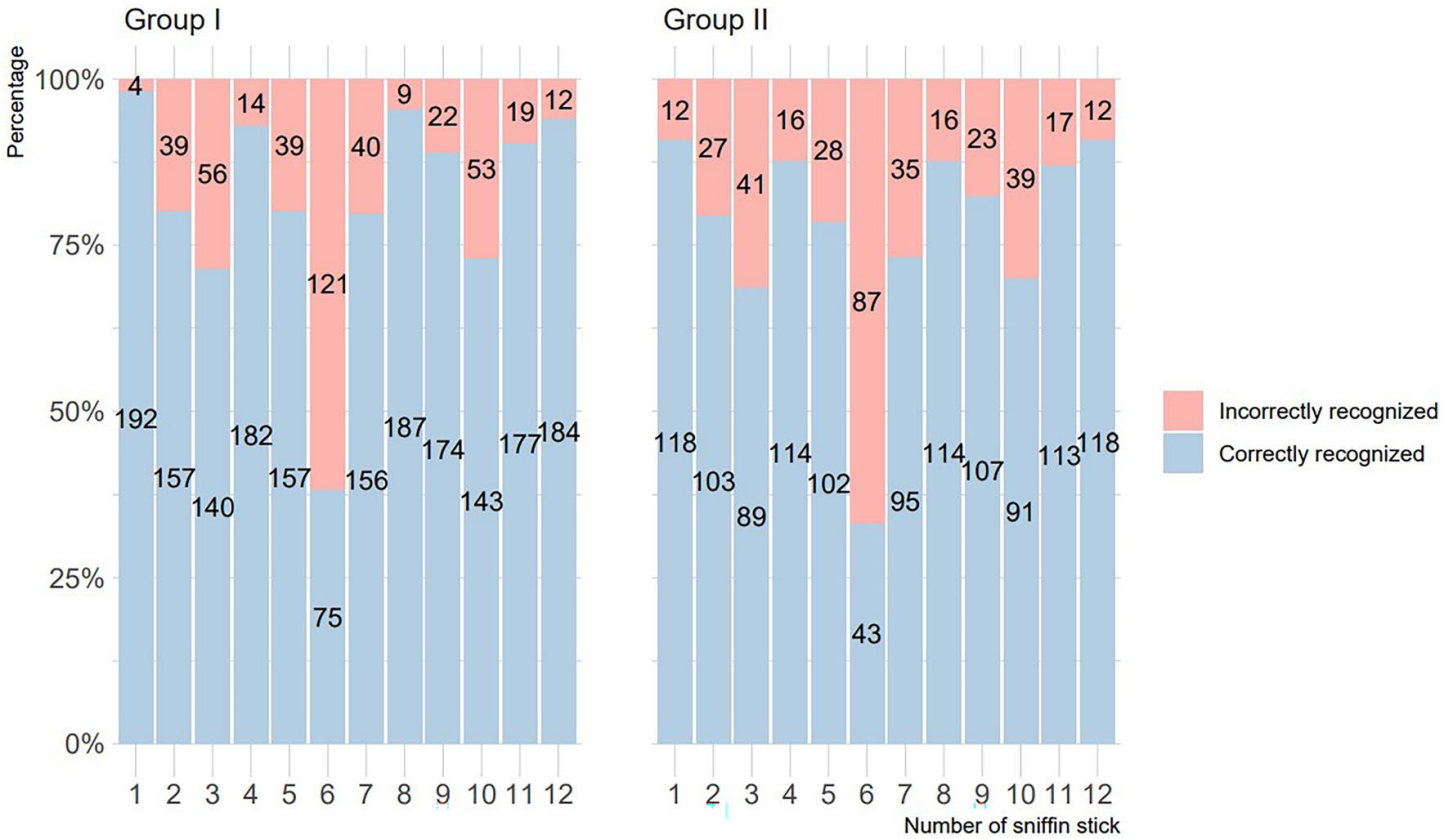
Graph of the correctly and incorrectly recognized sniffin sticks per group. Frequency shown as labels on bars. Group I is control sample and Group II is research sample.

196 patients from Group I received twelve fragrance samples which were the Sniffin Sticks Test. The fragrances in our study's samples are (in that order): 1. Orange; 2. Leather; 3. Cinnamon; 4. Mint; 5. Banana; 6. Lemon; 7. Licorice; 8. Coffee; 9. Cloves; 10. Pineapple; 11. Rose; 12. Fish. When evaluating each smell, individuals were given an answer card with four answers and marked the correct response according to them. First sniffin stick (orange) was correctly recognized by 192 of the 196 patients (if 1 is the correct answer and 0 is the incorrect answer, *x̅* = 0.98 ± 0.14). Second sample (leather) was correctly identified by 157 of the 196 individuals (*x̅* = 0.77 ± 0.42). Third sniffin stick (cinnamon) was correctly recognized by 140 of the 196 patients (*x̅* = 0.71 ± 0.45). Fourth sample (mint) was correctly identified by 182 of the 196 individuals (*x̅* = 0.93 ± 0.26). Fifth sniffin stick (banana) was correctly distinguished by 157 people (*x̅* = 0.8 ± 0.4). Sixth sample (lemon) was correctly recognized by 75 patients (*x* ® = 0.38 ± 0.49). Seventh sniffin stick (licorice) was correctly identified by 156 of the 196 individuals (*x̅* = 0.8 ± 0.4). Eighth sample (coffee) was correctly distinguished by 187 of the 196 patients (*x̅* = 0.95 ± 0.21). Ninth sniffin stick (cloves) was correctly recognized by 174 individuals (*x̅* = 0.89 ± 0.32). Tenth sample (pineapple) was correctly identified by 143 of the 196 people (*x̅* = 0.73 ± 0.45). Eleventh sniffin stick (rose) was correctly distinguished by 177 patients (*x̅* = 0.9 ± 0.3). Twelfth sample (fish) was correctly recognized by 184 of the 196 individuals (*x̅* = 0.94 ± 0.24). The results described above in addition to control sample are shown in Fig. 2.

130 patients from Group II received twelve fragrance samples which were the Sniffin Sticks Test. First sniffin stick (orange) was correctly recognized by 118 of the 130 patients (if 1 is the correct answer and 0 is the incorrect answer, *x̅* = 0.91 ± 0.29). Second sample (leather) was correctly identified by 103 of the 130 individuals (*x̅* = 0.79 ± 0.41). Third sniffin stick (cinnamon) was correctly recognized by 89 of the 130 patients (*x̅* = 0.68 ± 0.47). Fourth sample (mint) was correctly identified by 114 of the 130 individuals (*x̅* = 0.88 ± 0.33). Fifth sniffin stick (banana) was correctly distinguished by 102 people (*x̅* = 0.78 ± 0.41). Sixth sample (lemon) was correctly recognized by 43 patients (*x* ® = 0.33 ± 0.47). Seventh sniffin stick (licorice) was correctly identified by 95 of the 130 individuals (*x̅* = 0.73 ± 0.45). Eighth sample (coffee) was correctly distinguished by 114 of the 130 patients (*x̅* = 0.88 ± 0.33). Ninth sniffin stick (cloves) was correctly recognized by 107 individuals (*x̅* = 0.82 ± 0.38). Tenth sample (pineapple) was correctly identified by 91 of the 130 people (*x̅* = 0.7 ± 0.46). Eleventh sniffin stick (rose) was correctly distinguished by 113 patients (*x̅* = 0.87 ± 0.34). Twelfth sample (fish) was correctly recognized by 118 of the 130 individuals (*x̅* = 0.91 ± 0.29) (Fig. 2).

**Figure 3:**
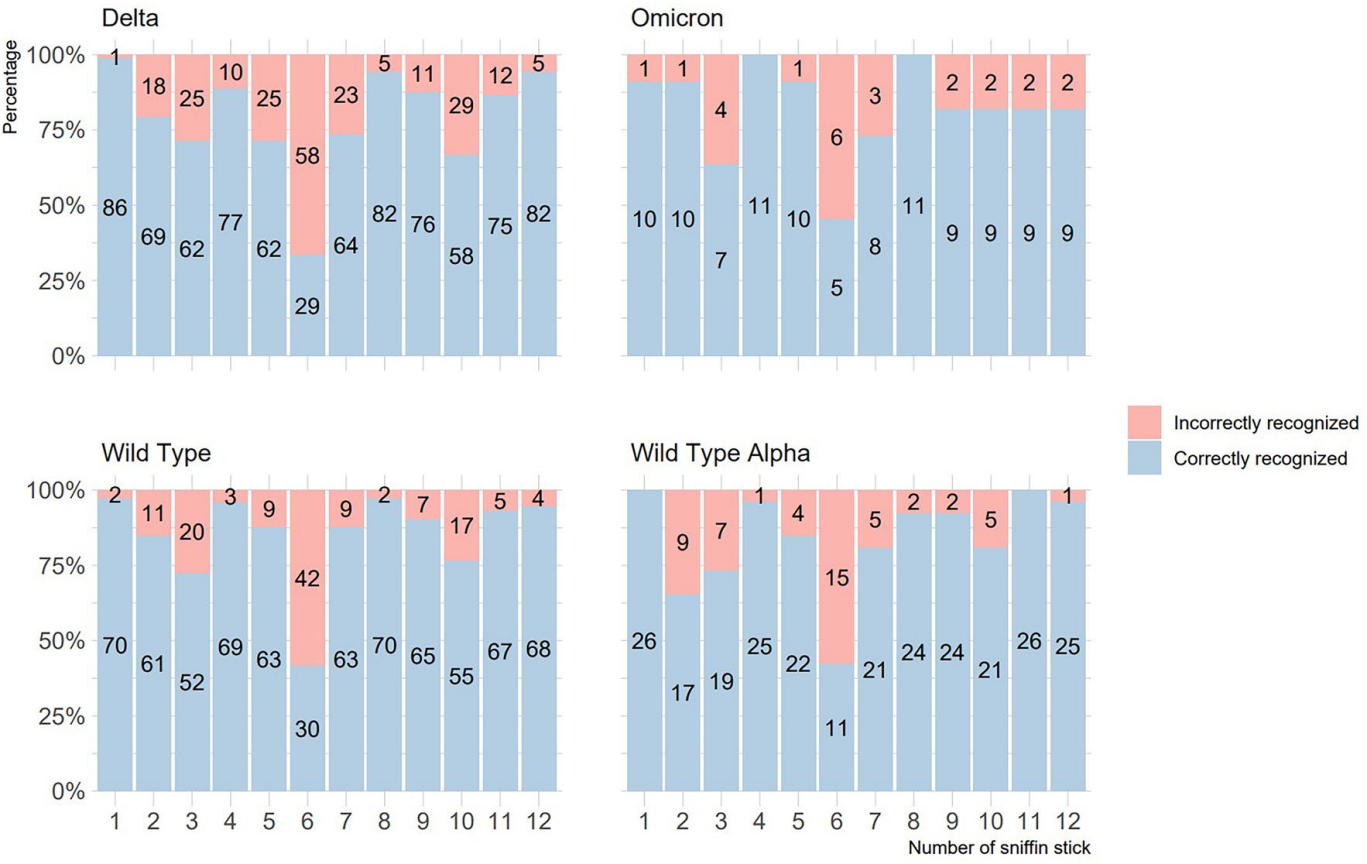
Graph comparing correctly and incorrectly recognized fragrances in research sample depending on the COVID-19 wave in which the patient was ill. Frequency shown as labels on bars.

In addition, the results of correctly recognized fragrances in research sample depending on the period of the COVID-19 pandemic in which the patient was ill are summarized in Fig. 3.

### Comparison of total score of the Sniffin Sticks Test between different waves in research sample

**Figure 4:**
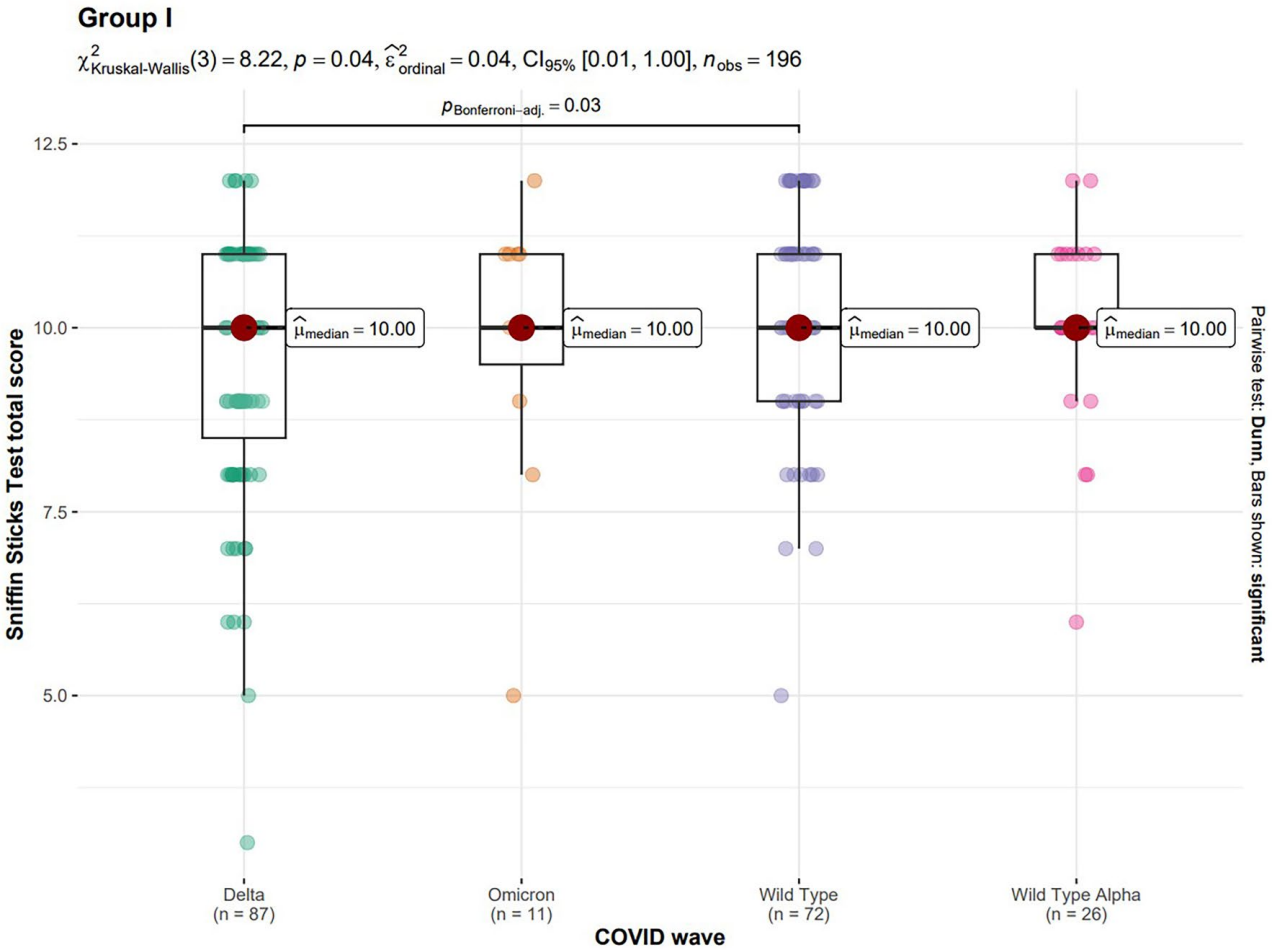
Comparison of total score of the Sniffin Sticks Test between Delta, Omicron, Wild Type and Wild Type Alpha waves in Group I.

It was also decided to make a comparison of total score of the Sniffin Sticks Test between Delta, Omicron, Wild Type and Wild Type Alpha waves. The particular waves consisted of: 87 people in Delta wave, 11 people in Omicron wave, 72 people in Wild Type wave and 26 people in Wild Type Alpha wave. Dunn's test with Bonferroni correction was used for this comparison. Only the difference between the Delta wave and the Wild Type wave turned out to be statistically significant. The results are shown in Fig. 4.

### Differences in recognizing particular smells in Group I

After assessment recognition of fragrances from the Sniffin Sticks data, differences in recognition of individual smells were compared. Dunn's test with Bonferroni correction was used for this comparison. Only statistically significant data (p-value < 0.05) were used for the comparison and differences in recognition of individual smells were confirmed. The greatest differences were observed in the comparison of the scents of orange and lemon (p.adj < 0.001; Z statistic = -15.313), lemon and coffee (p.adj < 0.001; Z statistic = 14.659), fish and lemon (p.adj < 0.001; Z statistic = 14.266), mint and lemon (p.adj < 0.001; Z statistic = 14.004) and rose and lemon (p.adj < 0.001; Z statistic = -10.069). Other statistically significant differences in the recognition of individual smells are demonstrated in Table 2.

**Table 2:** Differences in recognition of individual smells for Group I. The data have been ordered using absolute values of Z statistic, from highest to lowest.

Smell 1	Smell 2	Z statistic	*p* adjusted
Orange	Lemon	− 15.313	0.000
Lemon	Coffee	14.659	0.000
Fish	Lemon	− 14.266	0.000
Mint	Lemon	− 14.004	0.000
Rose	Lemon	− 13.350	0.000
Lemon	Cloves	12.957	0.000
Leather	Lemon	− 10.732	0.000
Banana	Lemon	− 10.732	0.000
Lemon	Licorice	10.601	0.000
Pineapple	Lemon	− 8.900	0.000
Cinnamon	Lemon	− 8.507	0.000
Orange	Cinnamon	− 6.806	0.000
Orange	Pineapple	− 6.413	0.000
Cinnamon	Coffee	6.151	0.000
Pineapple	Coffee	5.759	0.000
Fish	Cinnamon	− 5.759	0.000
Cinnamon	Mint	5.497	0.000
Pineapple	Fish	5.366	0.000
Pineapple	Mint	5.104	0.000
Rose	Cinnamon	− 4.843	0.000
Orange	Licorice	− 4.712	0.000
Orange	Leather	− 4.581	0.000
Orange	Banana	− 4.581	0.000
Pineapple	Rose	4.450	0.001
Cinnamon	Cloves	4.450	0.001
Pineapple	Cloves	4.057	0.003
Licorice	Coffee	4.057	0.003
Leather	Coffee	3.926	0.006
Banana	Coffee	3.926	0.006
Fish	Licorice	− 3.665	0.016
Fish	Leather	− 3.534	0.027
Fish	Banana	− 3.534	0.027
Mint	Licorice	− 3.403	0.044

### Correlation of total score of the Sniffin Sticks Test with various clinical data in Group I and Group II

During the Sniffin Sticks Test, a lot of clinical and laboratory data were evaluated, which were used to create a correlation of total score of the Sniffin Sticks Test with these clinical data. The Pearson correlation was used for this statistics. Statistically significant variables for control sample are summarized in Table 3 and for research sample in Table 4.

**Table 3:** Correlation of the mean of total points of the Sniffin Sticks Test of Group II with various clinical data.

Variable name	Pearson correlation coefficient	*p*
Geriatric Montreal Cognitive Assessment correction	0.27	0.004
Age	− 0.19	0.031
Troponin	− 0.37	0.001

**Table 4:** Correlation of the mean of total points of the Sniffin Sticks Test of Group I with various clinical data.

Variable name	Pearson correlation coefficient	*p*
Geriatric Montreal Cognitive Assessment correction	0.225	0.021
Geriatric Mini Mental State Examination (6 months after the disease)	0.195	0.044
Geriatric Montreal Cognitive Assessment (6 months after the disease)	0.193	0.047
LDL (6 months after the disease)	− 0.152	0.034
Creatinine (6 months after the disease)	− 0.173	0.016
Troponin (6 months after the disease)	− 0.213	0.013
Duration of the acute phase of the disease	− 0.218	0.017
Fasting serum glucose (6 months after the disease)	− 0.226	0.001
Geriatric Mini Mental State Examination correction	− 0.257	0.021
Age	− 0.258	0.000
Il-6 (6 months after the disease)	− 0.281	0.012

Moreover, qualitative variables evaluated during the Sniffin Sticks Test required statistical assessment in the Dunn test. Only the categorical variables of the research sample (Group I) turned out to be statistically significant and are presented in Table 5.

**Table 5:** Correlation of the dependent variable (Total score of the Sniffin Sticks Test) with the independent variables (sex, use of Dexaven and use of Ceftriaxone) in Group I.

Dependent variable	Independent variable 1	Independent variable 2	Number 1	Number 2	Statistic	*p*
Total score of the Sniffin Sticks Test	Sex: male	Sex: female	100	96	2.048	0.041
	Without Dexaven	Use of Dexaven	43	118	2.021	0.043
	Without Ceftriaxon	Use of Ceftriaxon	125	36	2.281	0.023

### Impact of various predictors on olfactory disorders during COVID-19 and on the result of the Sniffin Sticks Test in Group I

It was also decided to make models explaining the impact of various predictors on smell disorders during disease and on the result of the Sniffin Sticks Test. It turned out that the only statistically significant predictors in the case of olfactory disorders during COVID-19 were the Omicron wave and the Wild Type wave compared to the Delta wave. These results are presented in Table 6 and logistic regression was used to demonstrate them (Table 6).

**Table 6:** Logistic regression model of smell disorders during COVID-19 in Group I.

Logistic regression–**s**mell disorders during COVID-19				
Predictors	Odds Ratios	std. Error	CI	*p*
Age	0.98032	0.01432	0.95209–1.00848	0.174
Sex [female]	0.69549	0.2476	0.34447–1.39771	0.308
**COVID-19 Wave [Omicron]**	**0.228**	**0.16865**	**0.04536–0.90201**	**0.046**
**COVID-19 Wave [wild type]**	**0.31678**	**0.13434**	**0.13478–0.71630**	**0.007**
COVID-19 Wave [wild type Alpha]	0.57661	0.29862	0.20619–1.60255	0.288
Dexaven^1^	1.0086	0.42606	0.43698–2.31042	0.984
Ceftriaxon^1^	0.84201	0.36811	0.35495–1.98828	0.694
Observations	154			
R^2^ Tjur	0.096			

It is also worth noticing that in the case of the Quasi-Poisson Regression model (Table 7), the only statistically significant variables were sex (Incidence Rate Ratio = 1.068; Standard error = 0.029; Confidence interval = 1.012 – 1.127; P value = 0.016) and age (Incidence Rate Ratio = 0.996; Standard error = 0.029; Confidence interval = 0.993 – 0.998; P value < 0.001) in correlation with the dependent variable—the result of the Sniffin Sticks Test.

**Table 7:** Quasi-Poisson model of Sniffin Sticks Test total score in Group I.

Sniffin Sticks total score				
Predictors	Incidence Rate Ratios	std. Error	CI	*p*
**Age**	**0.99597**	**0.00111**	**0.99379**− **0.99815**	** < 0.001**
**Sex [female]**	**1.068**	**0.02911**	**1.01241–1.12660**	**0.016**
COVID-19 Wave [Omicron]	1.00382	0.05427	0.90181–1.11475	0.944
COVID-19 Wave [wild type]	1.05508	0.03455	0.98934–1.12484	0.102
COVID-19 Wave [wild type Alpha]	1.06321	0.0429	0.98179–1.15006	0.129
Dexaven^1^	0.97731	0.03109	0.91841–1.04040	0.471
Ceftriaxon^1^	0.95132	0.03247	0.88948–1.01683	0.144
**S**mell disorders during COVID-19^1^	0.96507	0.02632	0.91484–1.01805	0.192
Observations	154			
R^2^ Nagelkerke	0.182			

### Assessment of the severity of COVID-19 infection in Group I

**Figure 5:**
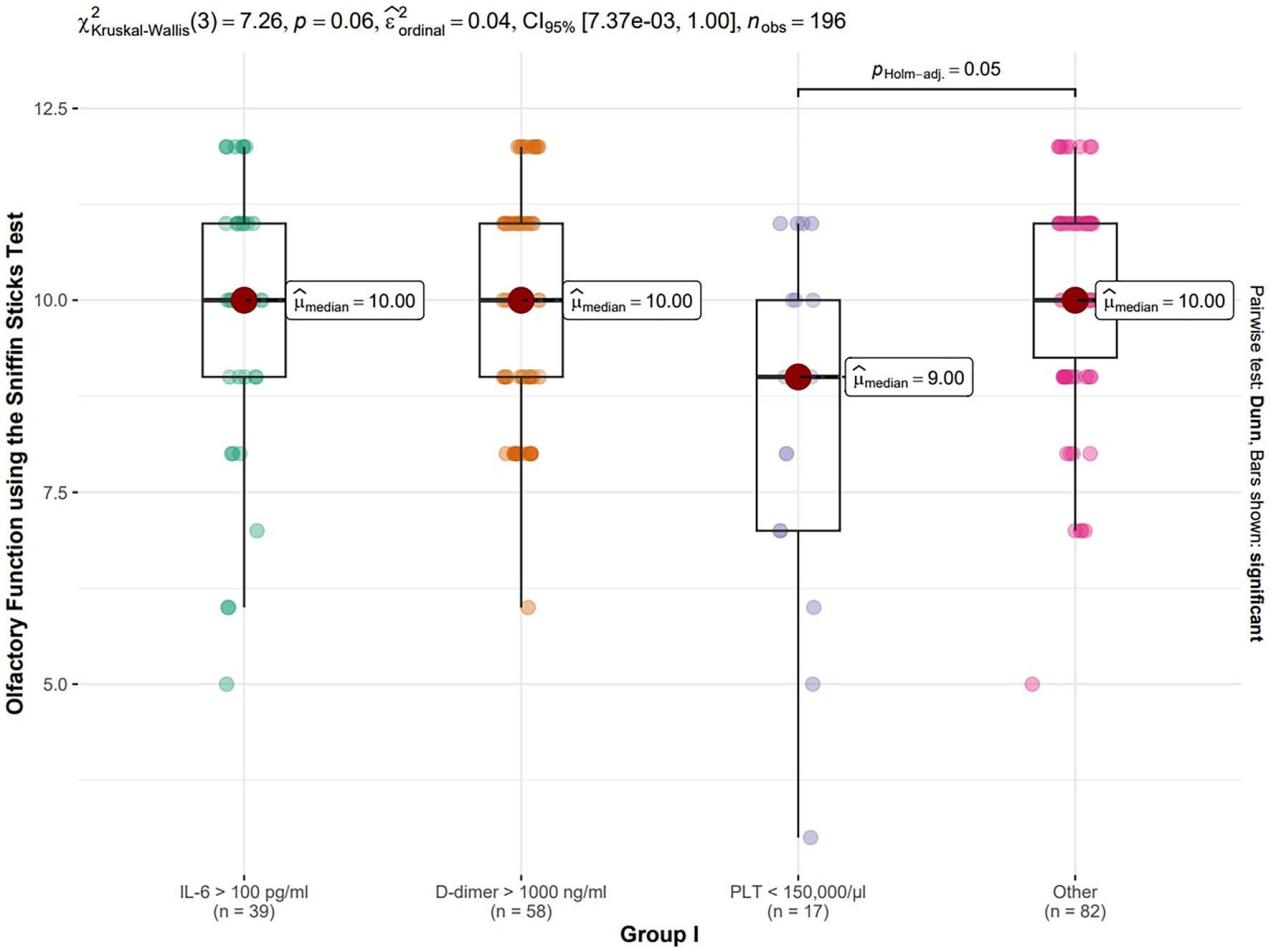
Assessment of the severity of COVID-19 infection in Group I.

Analysis of associations between COVID-19 severity assessed on laboratory parameters (IL-6 > 100 ​pg/ml, D-dimer concentration over 1000 ​ng/ml and PLT count below 150 000/μl) and persistence of smell loss showed statistically significant differences when PLT was examined^24^. Patients with PLT count below 150 000/μl had greater olfactory disorders than those with PLT count over 150 000/μl. The relation of smell disorders with severity of COVID-19 classified based on laboratory criteria abnormalities during COVID-19 is presented on Fig. 5.

## Discussion

Olfactory impairment is connected with lower quality of life, depression, diminished food satisfaction, inability to recognize dangerous environmental hazards, and decreased social well-being^25,26^. It has also been associated with increased mortality in elderly people^27^.

The mean age of the patients in research sample who participated in our study was 53.7 ± 12.6 years. These results are consistent with previous research indicating a connection between persistent olfactory loss and middle and older age^11,14^. COVID-19-related smell disorders rarely occur in either extreme age and are most popular in the 40–50-year-old age bracket^28,29^. There is correlation between age category and volume of expression of angiotensin-converting enzyme 2 (ACE2) receptors as well as other entry proteins or sustentacular cells^28,29^. For the middle-age group, the greatest volume of ACE2 expression was demonstrated, and nasal gene expression of ACE2 was found to raise with age (between 4 and 60 years old)^28,29^.

The nasal cavity plays an essential role in COVID-19 because it is an area of viral replication and one of the entry ways for SARS-CoV-2. SARS-CoV-2 appears to have its own ways of aggression to the nasal neuroepithelium, with a preference for neural implication over the mucous membrane in the nose^30,31^. Olfactory impairment in COVID-19 patients has been studied in research that assessed the symptom subjectively^10,32,33^. In COVID-19, the prevalence of self-reported smell impairment varies greatly between those studies, ranging from 23.7% to nearly 90%^9,31–33^. Subjective techniques of evaluation, on the other hand, are prone to a variety of biases and result in significant inaccuracy^34,35^.

Moreover, the amount of people with persistent olfactory loss described in this research is comparable to previous estimates, with reported rates of persistent smell loss at 6 months ranging from 4.7 to 27%^11–15^.

Dexamethasone and Ceftriaxone demonstrated negative correlations with the mean of total points of the Sniffin Sticks Test. This is due to the fact that these drugs were given to patients with severe COVID-19. Dexamethasone has been used extensively for cytokine storm during COVID-19 and late phase of this disease, while Ceftriaxone has been used empirically in patients with suspected bacterial superinfection during COVID-19^36,37^. Additionally, inflammatory parameters such as IL-6 also showed negative correlations with the mean of total points of the olfactory test. It was already proven that inflammatory parameters, such as IL-6 reflect the severity of the disease^24^. Also, the male sex influenced on the smell disturbances. It mirrors the differences in the disease course between the gender^38^. These reports confirm the results of our study.

It is also worth mentioning that the factors influencing the long-COVID syndrome (which consists of i.a. olfactory and taste disorders) are infections with former SARS-CoV-2 variants: Wild Type variants, Wild Type Alpha variants and Delta variants^39^. Furthermore, Omicron variants might be associated with a lower risk of developing long-COVID syndrome^39^. This was confirmed by Boscolo-Rizzo et al. which showed the prevalence and the severity of smell and taste disorders after COVID-19 has dropped significantly with the advent of the Omicron variant^40^. The most striking difference was observed for loss of sense of smell, a pathognomonic feature of earlier waves of SARS-CoV-2 infection, in Omicron wave presented in less than 20% of cases^41^. Interestingly, the two symptoms that were consistently more prevalent among Omicron than among Delta cases (regardless of vaccination status) were sore throat and hoarse voice^42^.

People with smell impairment may compensate by relying on other senses and recalling cognitive memories of particular fragrances^43^. It is expected that COVID-19 would cause chronic smell impairment in hundreds of thousands of people^44^. This research gives helpful data about short-term olfactory loss, allowing physicians to provide correct anticipatory guidance to patients. In our opinion, future research should focus on testing modifications to existing ways of treatment as well as the development of new therapies for smell disorders.

Interestingly, Petrocelli et al. observed a two-month asymptote after which there was little recovery. They proposed that the two-month period is a temporal threshold at which it was sensible to start empiric treatment^11^. Therefore, we think that the limitation of our study is the absence of performance of the Sniffin Sticks Test also during the disease and two months after the disease to compare the results at the same timepoint. Furthermore, other limitations are: the lack of measures of phantosmia and parosmia, which are main components of COVID-19 smell impairment, and the high number of subjective factors in this study so our results cannot be directly generalized to all individuals with a post-COVID olfactory disorder and should be verified in a larger prospective cohort study. However, our results showed, that patients after COVID-19 had higher tendency to smell disturbances (which may be a sign of neurodegeneration of olfactory nerves), even if patients were relatively younger.

To sum up, in the present study, we assessed smell disturbances 6 months after COVID-19 in Polish population. A large majority of patients recovered olfactory function within six months of COVID-19 onset assessed by Sniffin Sticks Test. The findings of this research might be used to inform individuals about the chance of recovery of smell after COVID-19 and estimation of predictors of smell disturbances after the COVID-19.

## Conclusions

1. There are smell differences between post-COVID-19 patients and healthy population.
2. There was statistically significant difference between the Delta wave and the Wild Type wave in Post-COVID-19 group in the total score of the Sniffin Sticks Test.
3. The smell disturbances depend on the age, cognitive impairments, clinical characteristics of the COVID-19 disease and sex of the patient.

## Data Availability

Data will be available on request by the Corresponding Author (as the research project is still ongoing).
